# Spread of injectate in ultrasound-guided serratus plane block: a cadaveric study

**DOI:** 10.1186/s40981-018-0147-4

**Published:** 2018-01-22

**Authors:** Tatsuya Kunigo, Takeshi Murouchi, Shuji Yamamoto, Michiaki Yamakage

**Affiliations:** 1 Department of Anesthesia, Hokkaido Medical Center for Child Health and Rehabilitation, 1-240-6, Kanayama 1-jo, Teine-ku Sapporo-shi, Hokkaido 006-0041 Japan; 2 Department of Anesthesia, Kitami Red Cross Hospital, Kitami, Japan; 3 Department of Anesthesia, Hokkaido P.W.F.A.C. Obihiro Kosei General Hospital, Obihiro, Japan; 4 Department of Anesthesiology, Sapporo Medical University School of Medicine, Sapporo, Japan

**Keywords:** Breast surgery, Cadaveric study, PECS block, Serratus plane block

## Abstract

**Background:**

Serratus plane block is a thoracic truncal block that has been proposed as alternatives for analgesia such as epidural anesthesia and paravertebral block for the anterolateral chest wall. Previously, we performed the clinical study about optimal volume of the local anesthetic in serratus plane block. The primary aim of this study was to assess the pattern of distribution of dye into the serratus plane of cadavers after ultrasound-guided serratus plane injection.

**Findings:**

Ultrasound-guided serratus plane injection was performed at the level of the fourth rib on the mid-axillary line in nine adult Thiel-embalmed cadavers. In each cadaver, one side was injected with 20 ml of methylene blue dye and the contralateral side with 40 ml. Dissections of the thoracic walls were performed 20 min after the injection. The spread of the dye to intercostal nerves, lateral and medial pectoral nerves, long thoracic nerve, and thoracodorsal nerves was assessed. All T2–T5 intercostal nerves in the 40-ml group and all T3–T4 nerves in the 20-ml group were stained with the dye. A larger number of intercostal nerves was stained in the 40-ml group than that in the 20-ml group. Medial and lateral pectoral nerves were not frequently stained in either group.

**Conclusions:**

The range of craniocaudal spread of the injectate was wider in the 40-ml group than that in the 20-ml group after ultrasound-guided serratus plane injection in Thiel-embalmed cadavers.

## Introduction

Pectoral nerve (PECS) block and serratus plane block are performed to suppress the pain in the anterior thoracic wall. Blanco et al. suggested that PECS I and II blocks and serratus plane block can be performed with ultrasound guidance [1–3]. The local anesthetic is injected between the pectoralis major and minor muscles for PECS I block, between the pectoralis minor and serratus anterior muscles for PECS II block, and between the latissimus dorsi and serratus anterior muscles for serratus plane block. Although the local anesthetic is injected on serratus anterior muscles in both PECS II block and serratus plane block, the local anesthetic is injected to more dorsal side in serratus plane block than in PECS II block. Therefore, serratus plane block can anesthetize more number of intercostal nerves. These blocks might be effective as alternative analgesia for the anterior thoracic wall without causing the potential serious complications reported after PVB.

Previously, we performed the clinical research to investigate optimal volume of the local anesthetic in serratus plane block [4]. In this study, we investigated the spread of the injectate by performing serratus plane injection with color dye in Thiel-embalmed cadavers and subsequent dissection.

## Materials and methods

This observational study was approved by the institutional ethics committee of Sapporo Medical University School of Medicine (#26-1-7).

Nine Thiel-embalmed cadavers were prepared for the study. Serratus plane injection was performed in the Thiel-embalmed cadavers with methylene blue dye. A 6- to 15-MHz linear array ultrasound transducer (S-Nerve, FUJIFILM-SonoSite, Tokyo, Japan) was placed on the mid-axillary line at the level of the fourth intercostal space. The interfascial plane between the serratus anterior and the pectoralis major muscles (the serratus plane) was identified. A 22-gauge block needle (Stimuplex Ultra, B-Braun Medical Inc., Melsungen, Germany) was inserted at the cephalad edge of the probe and advanced in-plane until the serratus plane. Once the needle tip was confirmed to be in the correct position, 20 or 40 ml of methylene blue dye was injected into the plane. Twenty milliliters of dye was injected to the left side and 40 ml, to the other.

Dissection of the thoracic walls was performed 20 min after the injection. The intercostal nerves, medial and lateral pectoral nerves, the long thoracic nerve, and the thoracodorsal nerve were identified. The spread of the injectate was assessed by color-stained nerves.

### Results

Nine thiel-embalmed cadavers were prepared; all were over 80 years old, four females and five males. The detail profiles including height and weight were not measured.

**Fig. 1:**
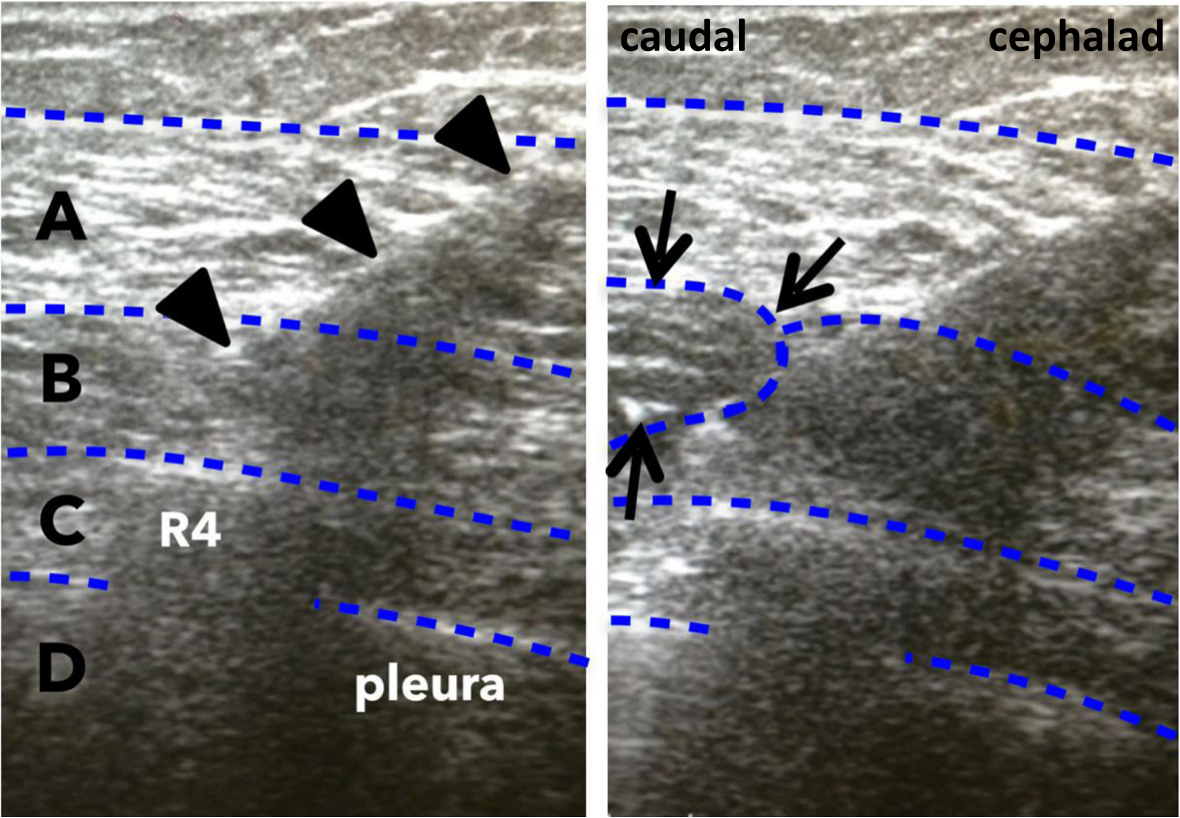
Ultrasound-guided serratus plane block

**Fig. 2:**
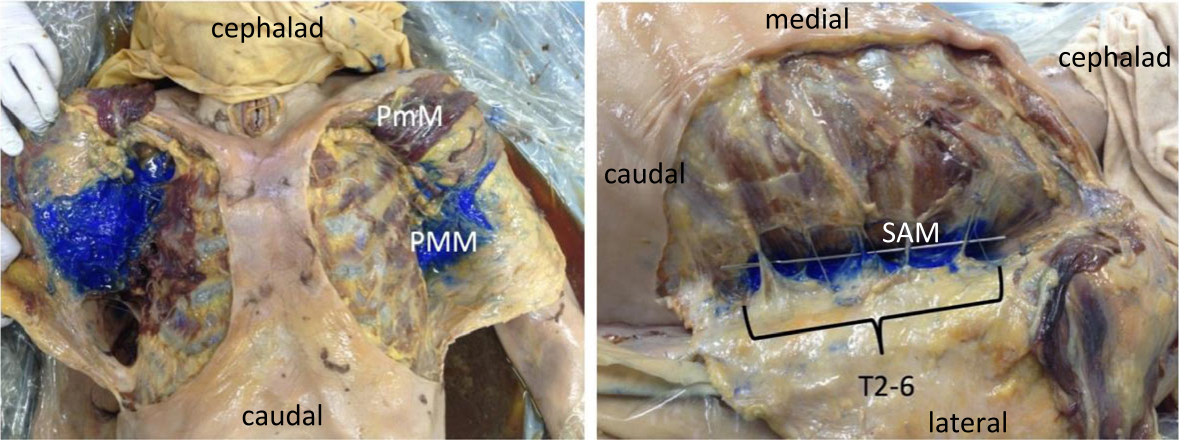
Dissections after color dye injections

One hemithoracic wall in the 40-ml group was excluded because of the surgical scar. The serratus planes, fourth ribs, needles, and spread of the dye were clearly visualized in all the other 17 specimens (Fig. 1). After the anterolateral chest walls had been dissected, the spread of the dye was confirmed at the surface of the serratus anterior muscle in all cases (Fig. 2). The intramuscular spread of the dye was not observed in dissection. All T2–T5 intercostal nerves in the 40-ml group and all T3–T4 nerves in the 20-ml group were stained by the dye (Table 1). The number of stained intercostal nerves was larger in the 40-ml group than that in the 20-ml group (5 ± 0.7 vs. 4 ± 1.1 nerves, *p* = 0.02). The degrees of spread to the medial and lateral pectoral, long thoracic, and thoracodorsal nerves were comparable between the groups.

**Table 1:** The number of nerves stained by dye

Affected nerve	20 ml (*n* = 9)	40 ml (*n* = 8)
T1	1	1
T2	3	8
T3	9	8
T4	9	8
T5	8	8
T6	3	5
T7	0	0
Lateral pectoral	2	3
Medial pectoral	1	3
Long thoracic	3	4
Thoracodorsal	2	4

Data are expressed as absolute numbers

T1–T7, corresponding intercostal nerves

### Discussion

This comparative study showed that 40 ml of the color dye spread to wider longitudinal directions after ultrasound-guided serratus plane injection in adult Thiel-embalmed cadavers than did 20 ml. Serratus plane block was reported to be effective in pain relief of the anterior chest wall [5, 6]. Besides, serratus plane block combined with PECS block was also reported to be effective [7–9]. T1 intercostal nerve and medial/lateral pectoral nerves were not frequently stained even when 40 ml of dye was used in our study. Long thoracic and thoracodorsal nerves were not frequently stained.

Blanco reported that sensory loss was obtained from T2 to T9 after injection of 0.4 ml/kg of 0.125% levobupivacaine for serratus anterior block [3]. The volume of injection in the present study, 40 ml, was larger than that in their study. Nevertheless, the range of spread of color dye in our study was narrower compared to the observed effects of sensory block in Blanco’s study. The difference could be because of the gap between clinical effect and anatomical evaluation of spread. Kikuchi et al. reported that the spread of the dye after PECS I and II blocks in a Thiel-embalmed cadaver extended to the mid-axillary line and to the surface of the serratus anterior muscle, respectively [10]. The result concerning serratus plane injection was similar to our study in that the dye spread over the anterolateral chest wall and in that the dye never spread into the axillary region. As mentioned above, T1 intercostal nerve, medial/lateral pectoral nerves, long thoracic nerve, and thoracodorsal nerve were seldom stained. The lateral pectoral nerve originates from C5–C7 and runs between the pectoral major and minor muscles. The medial pectoral nerve originates from C8–T1 and runs under the pectoral minor muscle. The long thoracic nerve originates from C5–C7 and runs on the serratus anterior muscle. The thoracodorsal nerve originates from C6–C8 and innervates the latissimus dorsi muscle, through the posterior part of the axilla. Our study showed that the dye injected into the serratus plane never reached the axilla or dorsal aspect and spread only over the anterolateral thoracic wall. PECS I block may be more suitable for blocking pectoral nerves [10].

Our study has several limitations. First, no anatomist verified the dissections. Second, the height and weight of cadavers were not measured. Finally, it is difficult to predict the clinical effect of a block in the cadaver research.

The dye spread was confirmed after serratus plane injection in Thiel-embalmed cadavers and subsequent dissections. The range of dye spread in the longitudinal direction was greater after injection of 40 ml than after 20 ml. However, a clinical study has shown that 20 and 40 ml were not significantly different for the analgesia after radical mastectomy [4]. The clinical analgesic effect cannot necessarily be reflected by the cadaveric result of the direct staining of individual nerve branches.
